# Supplementary material for: Bayesian multivariate longitudinal model for immune responses to Leishmania: A tick-borne co-infection study
Source: Stat Med. Author manuscript; Available in PMC 2024 May 24. (PMC11123579; doi:10.1002/sim.9837)
Supplement: Supplemental File 1 [file NIHMS1994131-supplement-Supplemental_File_1.pdf]

Supplemental Material 1: Simulation Study and Derivations of Full Conditionals

Bayesian Multivariate Longitudinal Model for Immune Responses to Leishmania - a tick borne Co-Infection Study

Felix M. Pabon-Rodriguez, Grant D. Brown, Breanna M. Scorza, Christine A. Petersen

1 Model and Derivation of Full Conditionals

1.1 Qualitative Categories for Disease Status

To assess the Canine Leishmaniasis (CanL) disease progression of subjects over time, we took into account LeishVet stage, pathogen load, and level of anti-*Leishmania* antibodies as explained in the paper. Each dog was classified based on the scoring proposed by Solano-Gallego et al. [1]. We further aggregate this scoring into the qualitative categories described below. For ith dog at time $t+1$, we define disease state $D_{i,t+1}$ as follows,

$$D_{i,t+1} = \begin{cases} 1\,(\text{Healthy}), & \text{if } LeishVet = 0 \text{ or } 1 \\ 2\,(\text{Mild}), & \text{if } LeishVet = 2 \\ 3\,(\text{Severe}), & \text{if } LeishVet = 3 \text{ or } 4 \\ 4\,(\text{Removed}), & \text{if removed due to severe case of Leishmaniasis.} \end{cases} \tag{1}$$

1.2 Bayesian Model

For $N = 50$ dogs and $T = 7$ time points, the proposed model is structured as follows

$$\begin{aligned} f_D(D_{i,t}, P_{i,t}, A_{i,t}, X_i) &= \left[\pi_{i,t}^{(1)}, \pi_{i,t}^{(2)}, \pi_{i,t}^{(3)}, \pi_{i,t}^{(4)}\right] \\ \pi_{i,t}^{(k)} &= \frac{exp\left[M_{i,t}^{D(k)}\boldsymbol{\beta}_D^{(k)} + X_i\boldsymbol{\alpha}_D\right]}{1 + \sum_{g=1,2,3} exp\left[M_{i,t}^{D(g)}\boldsymbol{\beta}_D^{(g)} + X_i\boldsymbol{\alpha}_D\right]} \\ \pi_{i,t}^{(4)} &= 1 - \pi_{i,t}^{(1)} - \pi_{i,t}^{(2)} - \pi_{i,t}^{(3)} \\ f_A(D_{i,t}, P_{i,t}, A_{i,t}, X_i) &= M_{i,t}^{A}\boldsymbol{\beta}_A + X_i\boldsymbol{\alpha}_A \\ f_P(D_{i,t}, P_{i,t}, A_{i,t}, X_i) &= M_{i,t}^{P}\boldsymbol{\beta}_P + X_i\boldsymbol{\alpha}_P \end{aligned} \tag{2}$$

$$\begin{aligned} D_{i,t+1} &\sim \text{Multinomial}\big(1; f_D(D_{i,t}, P_{i,t}, A_{i,t}, X_i)\big) \\ A_{i,t+1} &\sim \mathcal{N}\big(f_A(D_{i,t}, P_{i,t}, A_{i,t}, X_i),\, \sigma_A^2\big) \\ P_{i,t+1} &\sim \mathcal{N}\big(f_P(D_{i,t}, P_{i,t}, A_{i,t}, X_i),\, \sigma_P^2\big) \end{aligned} \tag{3}$$

for $k = 1, 2, 3$ in the probability terms, and $i = 1, \ldots, N$ and $t = 1, \ldots, T$.

1.3 Derivations

1.3.1 Complete Data Likelihood

Using the model definition as described by Equations 2 and 3, the joint likelihood can be defined by the product of each probability density (or probability mass) function corresponding to each model component. In this case, we have two continuous outcomes (pathogen load and antibody levels) and one categorical (disease status). To facilitate the definition of the likelihood presented in Equation 4, let us define $\boldsymbol{\theta}_P = (\boldsymbol{\beta}_P, \boldsymbol{\alpha}_P, \sigma_P^2)'$ as the set of vectors associated with pathogen load. Similarly, we can define the set of parameters associated with the antibody levels and disease status as $\boldsymbol{\theta}_A = (\boldsymbol{\beta}_A, \boldsymbol{\alpha}_A, \sigma_A^2)'$ and $\boldsymbol{\theta}_D = (\boldsymbol{\beta}_D^{(1)}, \boldsymbol{\beta}_D^{(2)}, \boldsymbol{\beta}_D^{(3)} \boldsymbol{\alpha}_D)'$, respectively.

$$
\begin{aligned}
\mathcal{L}(\theta_P, \theta_A, \theta_D | P, A, D) &= \prod_{i=1}^{N} \Big[f(P_{i,1}) \cdot f(A_{i,1}) \cdot f(D_{i,1}) \\
&\qquad \cdot \prod_{t=2}^{T} \Big\{ f(P_{i,t}|P_{i,t-1}, \theta_P) \cdot f(A_{i,t}|A_{i,t-1}, \theta_A) \cdot f(D_{i,t}|D_{i,t-1}, \theta_D) \Big\} \Big] \\
&\propto \prod_{i=1}^{N} \Bigg[f(P_{i,1}) \cdot f(A_{i,1}) \cdot f(D_{i,1}) \prod_{t=2}^{T} \Bigg\{ \Big(\frac{1}{\sigma_P} exp\Big\{ -\frac{1}{2\sigma_P^2}(P_{i,t} - \eta_{i,t-1}^{P})^2 \Big\} \Big) \\
&\qquad \cdot \Big(\frac{1}{\sigma_A} exp\Big\{ -\frac{1}{2\sigma_A^2}(A_{i,t} - \eta_{i,t-1}^{A})^2 \Big\} \Big) \\
&\qquad \cdot \Big({\pi_{i,t-1}^{(1)}}^{D_{i,t-1}^{(1)}} \cdot {\pi_{i,t-1}^{(2)}}^{D_{i,t-1}^{(2)}} \cdot {\pi_{i,t-1}^{(3)}}^{D_{i,t-1}^{(3)}} \cdot {\pi_{i,t-1}^{(4)}}^{1-\sum_{g=1}^{3} D_{i,t-1}^{(g)}} \Big) \Bigg\} \Bigg]
\end{aligned} \tag{4}
$$

Here we have that the mean expressions for each normal density distribution are defined as $\eta_{i,t-1}^{P} = M_{i,t}^{P}\boldsymbol{\beta}_P + X_i\boldsymbol{\alpha}_P$, and $\eta_{i,t-1}^{A} = M_{i,t}^{A}\boldsymbol{\beta}_A + X_i\boldsymbol{\alpha}_A$, respectively. The expressions for the $\pi_{i,t-1}^{(k)}$ are presented in Equation 2, and where $D_{i,t-1}^{(k)} = 1$ for $k = 1, 2, 3$ if $D_{i,t-1} = k$ and $D_{i,t-1}^{(k)} = 0$ otherwise, which are defined based in Equation 1. Note that a proportionality notation is used in the second line of the likelihood, which helps in factoring out all of the constants or fixed quantities. In addition, notice that we defined our baseline category in the multinomial distribution as $D_{i,t-1}^{(4)} = 1 - \sum_{g=1}^{3} D_{i,t-1}^{(g)}$.

1.4 Full Conditionals (Model Components)

Since pathogen load, antibodies level, and disease status were not observed for some of the time points, which are known as latent variables, then we estimated the corresponding components of the model for those time points. Therefore, the full conditional for the three main components of the model are given below.

1.4.1 Disease Status

$$
\begin{aligned}
f_c(D|P, A, \theta_P, \theta_A, \theta_D) \propto \prod_{i=1}^{N} \Bigg[f(P_{i,1}) f(A_{i,1}) f(D_{i,1}) \prod_{t=2}^{T} \Bigg\{ \Big(&{\pi_{i,t-1}^{(1)}}^{D_{i,t-1}^{(1)}} \cdot {\pi_{i,t-1}^{(2)}}^{D_{i,t-1}^{(2)}} \\
&\cdot {\pi_{i,t-1}^{(3)}}^{D_{i,t-1}^{(3)}} \cdot {\pi_{i,t-1}^{(4)}}^{1-D_{i,t-1}^{(1)}-D_{i,t-1}^{(2)}-D_{i,t-1}^{(3)}} \Big) \Bigg\} \Bigg]
\end{aligned} \tag{5}
$$

1.4.2 Pathogen Load

$$
f_c(P|A, D, \theta_P, \theta_A, \theta_D) \propto \prod_{i=1}^{N} \Bigg[f(P_{i,1}) f(A_{i,1}) f(D_{i,1}) \prod_{t=2}^{T} \Bigg\{ exp\Big\{ -\frac{1}{2\sigma_P^2}(P_{i,t} - \eta_{i,t-1}^{P})^2 \Big\} \Bigg] \tag{6}
$$

1.4.3 Antibodies Level

$$f_c(A|P,D,\theta_P,\theta_A,\theta_D) \propto \prod_{i=1}^{N}\Bigg[f(P_{i,1})f(A_{i,1})f(D_{i,1})\prod_{t=2}^{T}\Bigg\{exp\Big\{-\frac{1}{2\sigma_A^2}(A_{i,t}-\eta_{i,t-1}^{A})^2\Big\}\Bigg]\tag{7}$$

1.5 Full Conditionals (Parameters)

If ω_1 is equal to one of the parameters in $\{\beta_P,\alpha_P,\sigma_P^2\}$, then the full conditional is given by

$$f_c(\omega_1|.) \propto \prod_{i=1}^{N}\Bigg[f(P_{i,1})f(A_{i,1})f(D_{i,1})\prod_{t=2}^{T}\Bigg\{exp\Big\{-\frac{1}{2\sigma_P^2}(P_{i,t}-\eta_{i,t-1}^{P})^2\Big\}\Bigg\}\Bigg]\cdot\pi(\omega_1)\tag{8}$$

If ω_2 is equals to one of the parameters in $\{\beta_A,\alpha_A,\sigma_A^2\}$, then the full conditional is given by

$$f_c(\omega_2|.) \propto \prod_{i=1}^{N}\Bigg[f(P_{i,1})f(A_{i,1})f(D_{i,1})\prod_{t=2}^{T}\Bigg\{exp\Big\{-\frac{1}{2\sigma_A^2}(A_{i,t}-\eta_{i,t-1}^{A})^2\Big\}\Bigg\}\Bigg]\cdot\pi(\omega_2)\tag{9}$$

Finally, if ω_3 is equals to one of the parameters in $\{\beta_D^{(k)},\alpha_D\}$ for $k=1,2,3$, then the full conditional is given by

$$\begin{aligned}f_c(\omega_3|.) \propto \prod_{i=1}^{N}\Bigg[f(P_{i,1})f(A_{i,1})f(D_{i,1})\Bigg\{\prod_{t=2}^{T}\Big(&\pi_{i,t-1}^{(1)}{}^{D_{i,t-1}^{(1)}}\cdot\pi_{i,t-1}^{(2)}{}^{D_{i,t-1}^{(2)}}\cdot\pi_{i,t-1}^{(3)}{}^{D_{i,t-1}^{(3)}}\\ &\cdot\pi_{i,t-1}^{(4)}{}^{1-D_{i,t-1}^{(1)}-D_{i,t-1}^{(2)}-D_{i,t-1}^{(3)}}\Big)\Bigg\}\Bigg]\cdot\pi(\omega_3)\end{aligned}\tag{10}$$

In Equations 8-10, $\pi(\omega_1)$, $\pi(\omega_2)$, and $\pi(\omega_3)$ denote the prior distribution of the parameters associated with the specific model component. A complete list of these prior specifications is provided in the main text (Section 3.5 Computation and Model Diagnostics).

2 Simulation Study

In this section, we present a simulation study for the development and implementation of the within-host Bayesian modeling of *Leishmania* infection, but the model presented here is a more straightforward case than the one presented in the main article. In order to develop an effective protective immune response against pathogens, it is necessary to have some specific and complex coordinated immunological events happen over a specific period of time. These events involve a variety of immune cells, within different tissues or organs, and the interplay between the pathogen load in the host's system, the clinical signs of the subject, and these immune responses.

2.1 Infection States

The first step in this development is to propose a simple organizing principle to describe infection state as consisting of three high-level categories: regulatory and inflammatory responses from the immune system, and pathogen load. The regulatory and inflammatory processes have multiple components subdivided into innate and adaptive immune processes and potential phenomena related to particular cell types. While immunological assays are uniformly more granular than this proposed aggregate framework, we propose that these categories represent a meaningful infection-immune state and provide a useful way to describe the progression of these states. The pathogen state within a host is defined by the amount and composition of the infectious organisms.

2.2 Model Specification

The model for this exploratory work considers the fitting of longitudinal data on individual hosts and their clinical status. For one, we define $t_{i,j}$ to be the time index j of subject i, with the baseline value for an individual host denoted as $t_{i,1}$, where the time is modeled discretely. Let $P_{i,j}$ be a scalar in the single pathogen model context (pathogen burden of Canine Leishmaniasis), denoting the pathogen load for the corresponding indexes. We measure the inflammatory state of the adaptive immune response by characterizing CD4 cells and associated cytokines, including the concentration of T_H1 cells, as well as the level of IFN-γ being expressed. We also include measures of antibody expression by B-cells. We denote the latent inflammatory state of an immune response as column vector $I_{i,j}$, with elements varying by the pathogen. For example, we might define $I_{i,j}$ to be a two column-vector where the first component, $I_{i,j,1}$, captures the T-cell-related inflammatory response, and the second, $I_{i,j,2}$, captures the B-cell related antibody response. The regulatory response $R_{i,j}$ is closely coupled with the inflammatory parts of the adaptive immune system. For example, we can focus on the CD4 cells: serum concentration of T_H1-reg cells, IL-10 expression, and cell-surface PD-1 expression. The latent regulatory state of an immune response might be a two column-vector as well, $R_{i,j}$, for the appropriate indices, and similar to the inflammatory specification, it could comprise T-cell regulatory response and B-cell related antibody regulatory response.

For all of the described state components, we assume that the future state is expected to depend on the current state, as well as possibly on the case history. In order to capture this temporal dependence, we introduce the arrays $R_{i,t<j+1}$, $I_{i,t<j+1}$, and $P_{i,t<j+1}$ to denote the latent regulatory, inflammatory, and pathogen state vectors for subject i from time $t_{i,1}$ to $t_{i,j}$. In Equation 11, we observe three unknown update functions for the regulatory (R) and inflammatory (I) responses of the immune system as well as for pathogen load (P), which describe the expected state at the next time point for each of the three main model components. In addition, these update equations describe individual-specific error components, capturing measurement error and individual heterogeneity. In general, the R and I components are allowed to depend on the entire response history of the host but change in pathogen burden P depends only on the current immunopathogenic state. The chosen form of these functions depends on pathogen-specific concerns and can range in complexity from straightforward linear functions of state components to involving interaction terms, and even to complex nonlinear expansions of the state vector.

$$\begin{aligned} R_{i,j+1} &= f_1(R_{i,t<j+1}, I_{i,t<j+1}, P_{i,t<j+1}) + \epsilon_{i,j}^{(1)} \\ I_{i,j+1} &= f_2(R_{i,t<j+1}, I_{i,t<j+1}, P_{i,t<j+1}) + \epsilon_{i,j}^{(2)} \\ P_{i,j+1} &= f_3(R_{i,j}, I_{i,j}, P_{i,j}) + \epsilon_{i,j}^{(3)} \end{aligned} \tag{11}$$

For the simulation study, we define the following notations of model components,

- $I_{i,j,1}$ to denote the proportion of (interferon) IFNγ+ CD4+ T cells,
- $I_{i,j,2}$ the proportion of proliferative CD4+ T cells,
- $R_{i,j,1}$ the proportion of (interleukin) IL10+ CD19+ B cells,
- $R_{i,j,2}$ the proportion of (interleukin) IL10+ CD4+ T cells, and
- P_{ij} the number of parasites per 10^6 peripheral blood mononuclear cell (PBMC)

In real datasets, these measurements could be obtained from different diagnostic assays from the laboratory, such as the enzyme-linked immunoassay (ELISA), real-time quantitative polymerase chain reaction (RT-qPCR), SNAP 4Dx Plus test, and among others.

2.2.1 Assumptions

In most cases when sample extraction is performed, it is not usually known the exact amount of pathogen load that is initially eliminated by the innate immune system. Therefore, we have addressed

this by assuming that the pathogen load measured at the first time point is the pathogen load after the innate immune system has eliminated a certain amount of the pathogen genetic material, and it is not a measure of the pathogen burden that was introduced initially by the infectious agent in the host.

2.2.2 State Transitions and Rules

To define clinical status and progression, we defined three categories of disease state, based primarily on pathogenic information. We say that a subject could be healthy (H) or in a silent state; be asymptomatic or in an acute state where inflammatory responses (I) are mainly activated; or in a symptomatic or chronic state, where regulatory (R) activation occurs. Therefore, to determine the transitions between each of the disease states, we used the work from Esch et al. [2] as a basis to define the following transition rules for disease state:

1. A subject is in healthy state (H) if pathogen load (P) is less than 10 parasites/10^6 PBMCs.
   Remain in H if $P < 10$

2. A subject transitions to inflammatory state (I) if pathogen load (P) is greater or equals than 10 parasites/10^6 PBMCs, but less than 100 parasites/10^6 PBMCs.
   Transition from H to I if $10 \leq P < 100$

3. A subject transition to regulatory state (R) if pathogen load (P) is greater or equals than 100 parasites/10^6 PBMCs.
   Transition from I to R if $P \geq 100$

Based on this set of rules, we can now define the transition functions and probabilities of state transitions, which are the basis for the construction of the simulated data set. In general, it is common to see an increase in pathogen load earlier in the course of an infection. Once the inflammatory responses of the immune system are activated by some specific cells, we can possibly observe a slight decline in the pathogen load, which is subject-specific. If treatment is not considered during the course of infection and the inflammatory response is not effectively fighting the disease, the pathogen load is expected to increase again, causing progression to the worst state, previously denoted as the chronic state. Figure 1 of Petersen [3], shows a temporal trend for parasite load as well as for CD4+ T cells, and productive versus non-productive antibodies. Using this information on the temporal behavior of a common trend in pathogen load, we were able to define transition functions for the state components of the CanL infection that can provide this behavior for pathogen load.

2.2.3 Example: Random Walk

As an example, we run a random walk simulation by using all of the information explained in previous sections to show how subjects can transition among disease state space based on immunopathogenic information. For instance, a random walk for the interplay of VL pathogen load and clinical state for a single individual is shown in Figure 1, where the threshold values used to define the transition rules are being plotted as horizontal dashed lines. The plot shows how disease classification changes as a function of pathogen load. Notice that for this particular artificial subject, we were able to identify the disease states over time. The subject started in the healthy state but it transition to the second state once it crossed the first cutoff value as presented in the second rule as presented in Subsection 2.2.2. Usually, when the inflammatory response is activated, we can see a slight decline in the pathogen load, which in Figure 1 starts from time point 2. Then, if treatment is not considered, as we assumed in this simulation setting, and if the inflammatory response is not effectively eliminating the disease material, then pathogen load is expected to increase causing a progression to the worst state, the regulatory state, as we observed for this particular subject towards the end of the temporal trend.

For the case of the inflammatory and regulatory responses, we used previous works from the literature to simulate corresponding values. For instance, Schaut et al. [4] provided that approximate percentages of IFNγ+ CD4+ T cells was fluctuating from 10% to 35% during the progression from a healthy to a regulatory state. The same authors also provided guided values for the percentage of

proliferative CD4+ T cells ranging from 15% to 50%, the percentage of IL10+ CD19+ B cells ranging from 20% to 65%, and the percentage of IL10+ CD4+ T cells ranging from 3% to 25%. On the other hand, guided values for pathogen load (Canine Leishmaniasis) were provided by Esch et al. [2], where the number of parasites/10^6 PBMCs greater than 100 corresponds to a subject in a chronic state. If we replicate the random walk process for several subjects, we obtain the temporal trends in Figure 2, where the red line represents the mean pathogen load over time. In this setting, we assume that all data was observed, but in real studies, this is usually not the case.

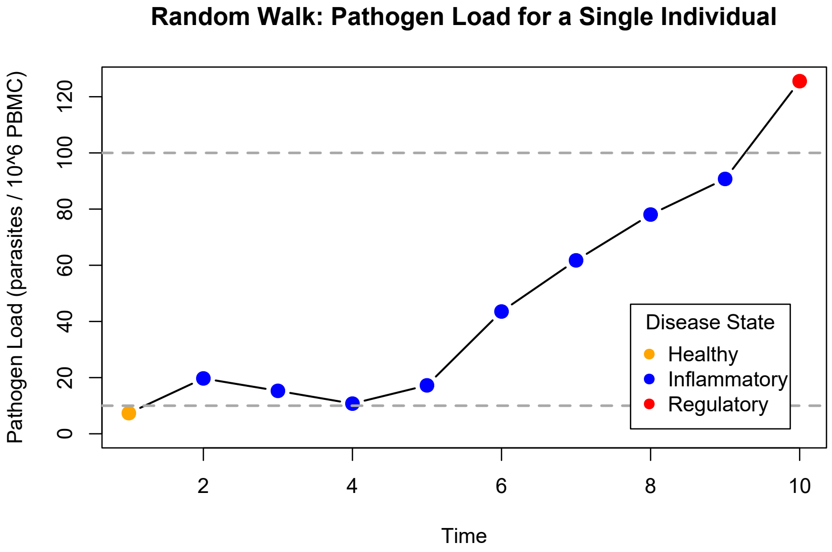

Figure 1: **Random Walk.** Pathogen load trajectory for a single individual where disease progression is classified based on transition rules as defined in Subsection 2.2.2.

2.3 Bayesian Model

Now that we have discussed the initial stages of the exploratory work, which was the simulation of the data set, we can now define the Bayesian model that is being proposed. We now assume that the generating process of the simulated data is not known, in particular, we are assuming that the nonlinear functions that were used in simulating the data are not known as well. The Bayesian model is defined as in Equation 12. In this specification, $g_A(A = \{P, I, R\})$ represents a basis-spline expansion over the range of the latent variables, and C_{ij} (optionally) denotes a relevant individual specific covariate vector. We also include interactions between the operands (Int). The terms collected into X^1_{ij} and X^2_{ij} form the overall design vectors for each component for subject i at time j. This highly flexible specification is designed to allow us to learn previously unknown nonlinear effects of the current state on future developments, including effects like plateaus, polynomial-like relationships, and possible interactions.

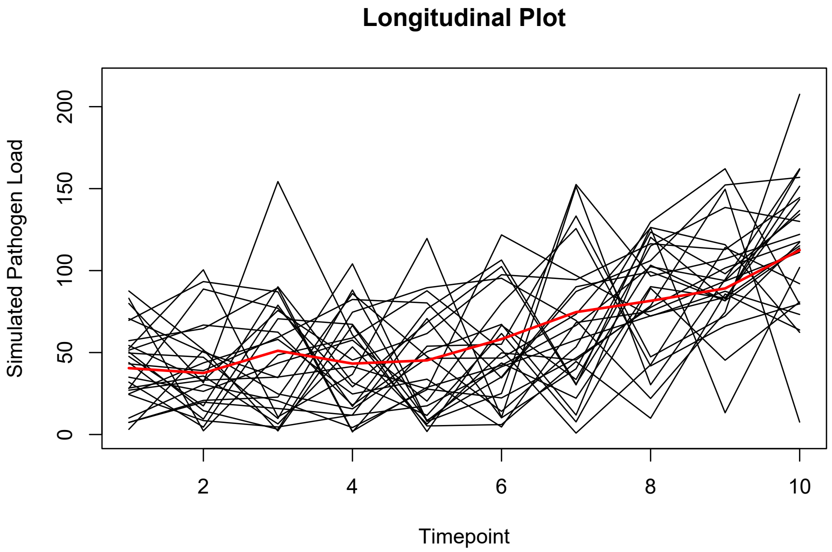

Figure 2: **Random Walk Replication.** Pathogen load trajectories for 50 individuals where disease progression is classified based on transition rules defined in Subsection 2.2.2. The red line indicates the group means over time.

$$
\begin{aligned}
I_{i,j+1,1} &\sim N(\eta_1^{(2)}, \sigma_{I_1}^2) \\
I_{i,j+1,2} &\sim N(\eta_2^{(2)}, \sigma_{I_2}^2) \\
\eta_1^{(2)} &= X_{ij}^3 \boldsymbol{\beta}^{(3)} + v_{12} \\
\eta_2^{(2)} &= X_{ij}^4 \boldsymbol{\beta}^{(4)} + v_{22} \\
X_{ij}^3 &= [1, g_R(R_{ij1}), g_I(I_{ij1}), g_I(I_{ij2}), Int, C_{ij}] \\
X_{ij}^4 &= [1, g_R(R_{ij1}), g_I(I_{ij2}), P_{ij}, Int, C_{ij}] \\
\\
R_{i,j+1,1} &\sim N(\eta_1^{(1)}, \sigma_{R_1}^2) \\
R_{i,j+1,2} &\sim N(\eta_2^{(1)}, \sigma_{R_2}^2) \\
\eta_1^{(1)} &= X_{ij}^1 \boldsymbol{\beta}^{(1)} + v_{11} \\
\eta_2^{(1)} &= X_{ij}^2 \boldsymbol{\beta}^{(2)} + v_{21} \\
X_{ij}^1 &= [1, g_R(R_{ij1}), g_I(I_{ij2}), Int, C_{ij}] \\
X_{ij}^2 &= [1, g_R(R_{ij1}), P_{ij}, Int, C_{ij}] \\
\\
P_{i,j+1} &\sim N(\eta^{(3)}, \sigma_p^2) \\
where &: \\
\eta^{(3)} &= X_{ij}^5 \boldsymbol{\beta}^{(5)} + v_3 \\
X_{ij}^5 &= [1, g_I(I_{ij1}), P_{ij}, Int, C_{ij}]
\end{aligned} \tag{12}
$$

2.3.1 Posterior Results and Diagnostics

We fit the model using the `rjags` package [5] in R [6, 7]. For the purpose of this simulation, we considered 25 hosts and 10 points in time. Three chains of 11,000 iterations with 1,000 as a burn-in period were used, for a total of 10,000 on each chain. Independent standard normal distributions were considered for the beta coefficients as well as for the between and within host variability. The gamma distribution is used for the precision parameter since the normal distribution in `rjags` is defined in terms of precision, which is the inverse of the variance. Posterior results of selected parameters are shown in Table 1, which are related to the pathogen component of the model. Notice here that all posterior means are positive, which indicates that the corresponding variable produces an increasing effect on the pathogen load in a future state. The column of the potential scale reduction factor, $\hat{R}$, tells us that selected parameters reached a level of convergence. The posterior density and trace plots are shown in Figure 3.

**Parameter**	**Mean**	**SD**	**2.5%**	**97.5%**	$\hat{R}$
$\beta_{5[1]}$	0.826	0.922	-1.041	2.624	1.001
$\beta_{5[2]}$	0.246	1.013	-1.747	2.166	1.001
$\beta_{5[3]}$	1.119	0.162	0.817	1.446	1.001
$\beta_{5[4]}$	0.304	0.451	-0.608	1.151	1.001
$\tau_P = 1/\sigma_P^2$	0.004	0.000	0.003	0.005	1.001
v_3	0.789	0.928	-1.003	2.607	1.002
Deviance	81.100	54.212	25.097	137.055	1.001

Table 1: **Results.** Posterior summary of the 3 MCMC chains for some selected model parameters. In this summary, posterior means, standard deviation, 95% credible interval are provided, and measures for convergence diagnostic are provided. Selected parameters are associated with pathogen load.

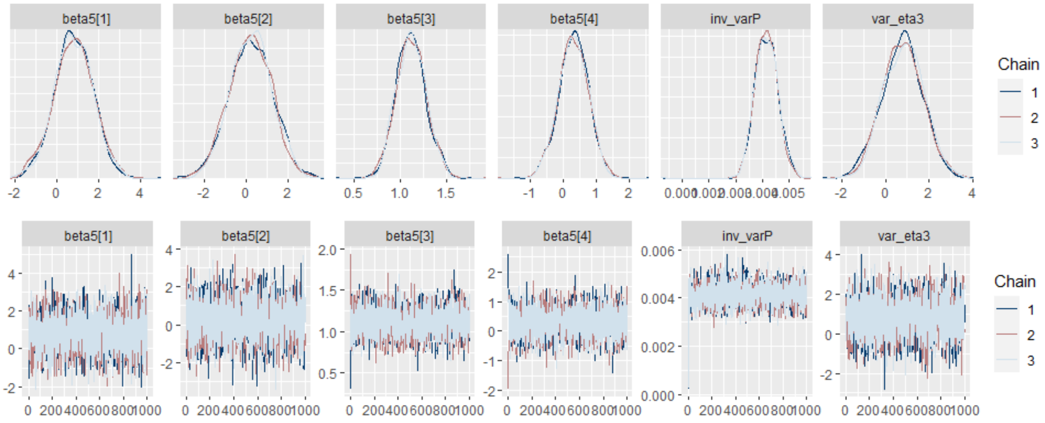

Figure 3: **Posterior Plots.** Posterior density and trace plots for the selected parameters associated with pathogen load.

In Figure 4, we present the (simulated) observed pathogen load trajectories with the posterior predicted pathogen load and corresponding 95% credible bands. From this plot, we observe how close the predicted data is to the observed curve. We estimated that the model explains 87% variability of the pathogen component, indicating good performance of the model.

2.4 Discussion

In the main article, we expand this model specification, and we repeat the analysis by using real longitudinal data, including clinical and molecular diagnostic data of a cohort of dogs that are naturally

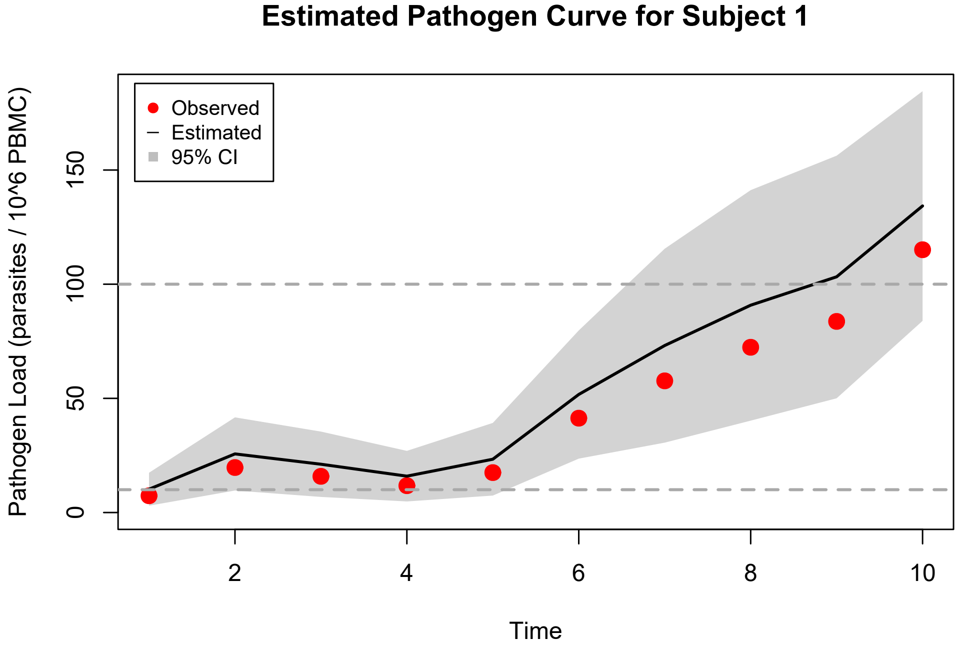

Figure 4: **Estimation.** Estimated pathogen load for a particular subject.

exposed to *L. infantum.* In addition, we incorporate individual-specific covariates such as age, clinical conditions such as indicators for co-infection, and treatment assignment. In addition to characterizing evidence for the processes driving disease progression, we predict individual and aggregate patterns of CanL progression, by using posterior predictive trajectories based on posterior distributions of the model parameters.

References

[1] Solano-Gallego L, Miró G, Koutinas A, et al. LeishVet guidelines for the practical management of canine leishmaniosis. *Parasites & vectors* 2011; 4(1): 1–16.

[2] Esch KJ, Juelsgaard R, Martinez PA, Jones DE, Petersen CA. Programmed Death 1–mediated T cell exhaustion during visceral leishmaniasis impairs phagocyte function. *The Journal of Immunology* 2013; 191(11): 5542–5550.

[3] Petersen CA. Leishmaniasis, an emerging disease found in companion animals in the United States. *Topics in companion animal medicine* 2009; 24(4): 182–188.

[4] Schaut RG, Grinnage-Pulley TL, Esch KJ, et al. Recovery of antigen-specific T cell responses from dogs infected with Leishmania (L.) infantum by use of vaccine associated TLR-agonist adjuvant. *Vaccine* 2016; 34(44): 5225–5234.

[5] Plummer M. rjags: Bayesian graphical models using MCMC. R package version 4-13. *Statistical software* 2022.

[6] R Core Team . *R: A Language and Environment for Statistical Computing.* R Foundation for Statistical Computing; Vienna, Austria: 2022.

[7] RStudio Team . *RStudio: Integrated Development Environment for R.* RStudio, PBC; Boston, MA: 2022.
