# Supplementary material for: Bayesian multivariate longitudinal model for immune responses to Leishmania: A tick-borne co-infection study
Source: Stat Med. Author manuscript; Available in PMC 2024 May 24. (PMC11123579; doi:10.1002/sim.9837)
Supplement: Supplemental File 2 [file NIHMS1994131-supplement-Supplemental_File_2.pdf]

Supplemental Material 2: MCMC Diagnostics

Bayesian Multivariate Longitudinal Model for Immune Responses to Leishmania - a Tick Borne Coinfection Study

Felix M. Pabon-Rodriguez, Grant D. Brown, Breanna M. Scorza, Christine A. Petersen

MCMC Diagnostics. The following R output provides the Gelman-Rubin diagnostic and traceplots for the model parameters considering the 3 full MCMC chains ($20,000$ iterations each) after $5,000$ iterations were discarded as burn-in. The Rhat value shown in the density plots (right side) represent the point estimate for the Gelman-Rubin diagnostic. The value 1.1 is used as a threshold, as explained in the main text. Therefore, we say that a factor of 1.1 or below indicates that the parameter has reached a level of convergence, which was achieved by all parameters in the model.

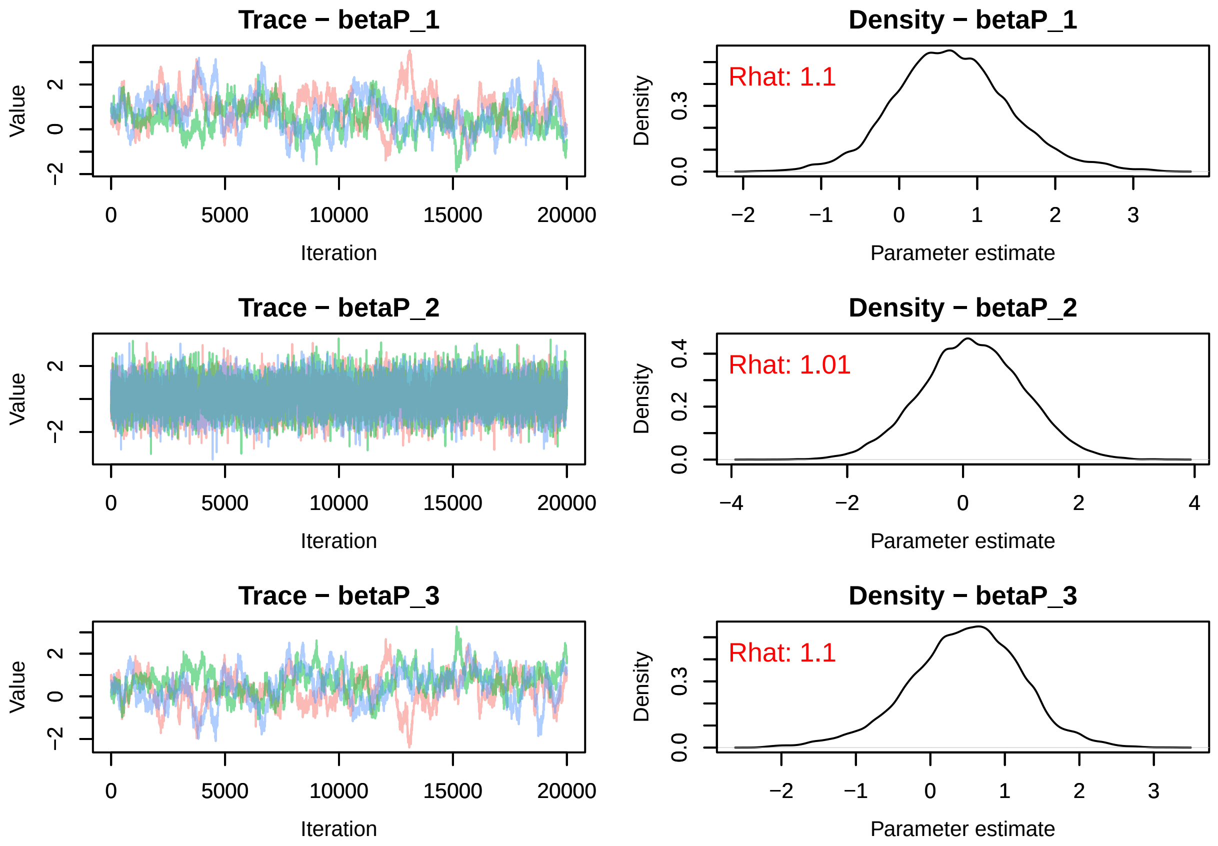

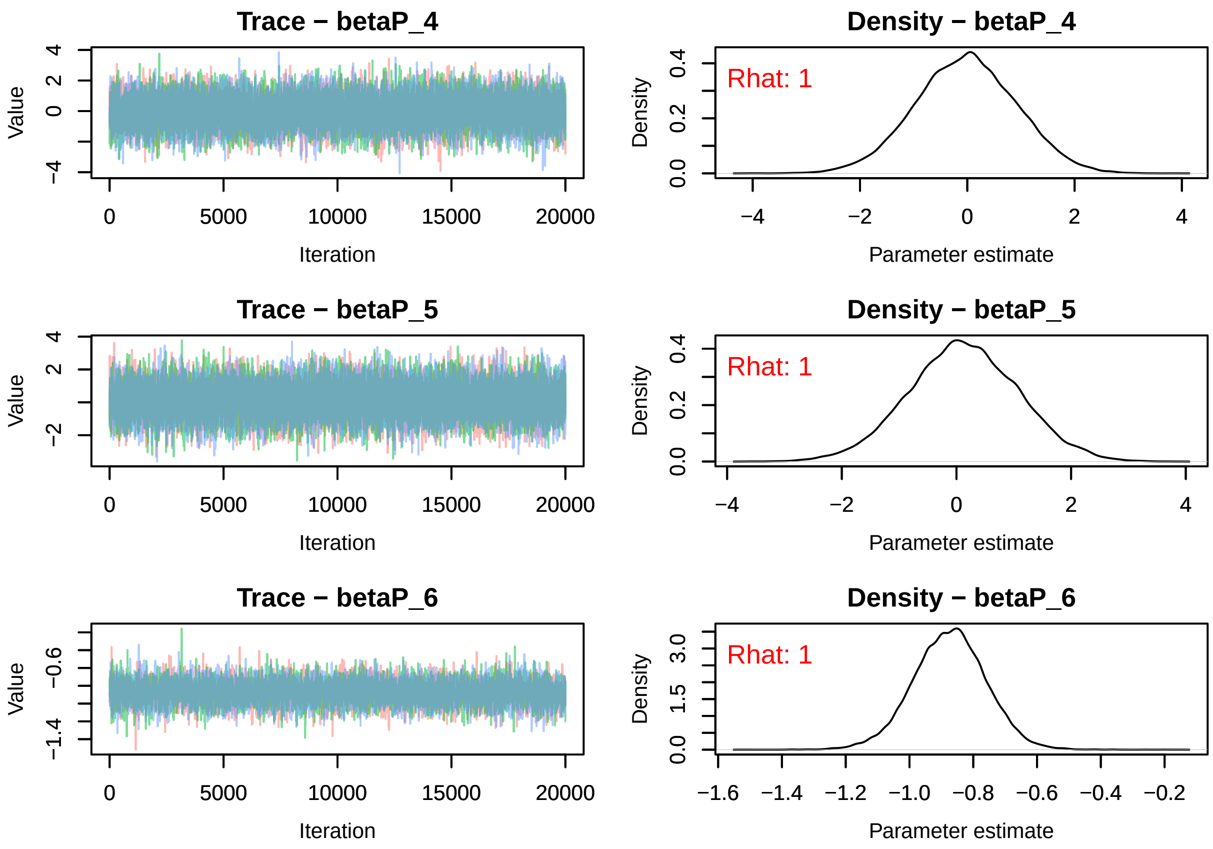
Trace − betaP_4
Value
Iteration
Density − betaP_4
Rhat: 1
Density
Parameter estimate
Trace − betaP_5
Value
Iteration
Density − betaP_5
Rhat: 1
Density
Parameter estimate
Trace − betaP_6
Value
Iteration
Density − betaP_6
Rhat: 1
Density
Parameter estimate

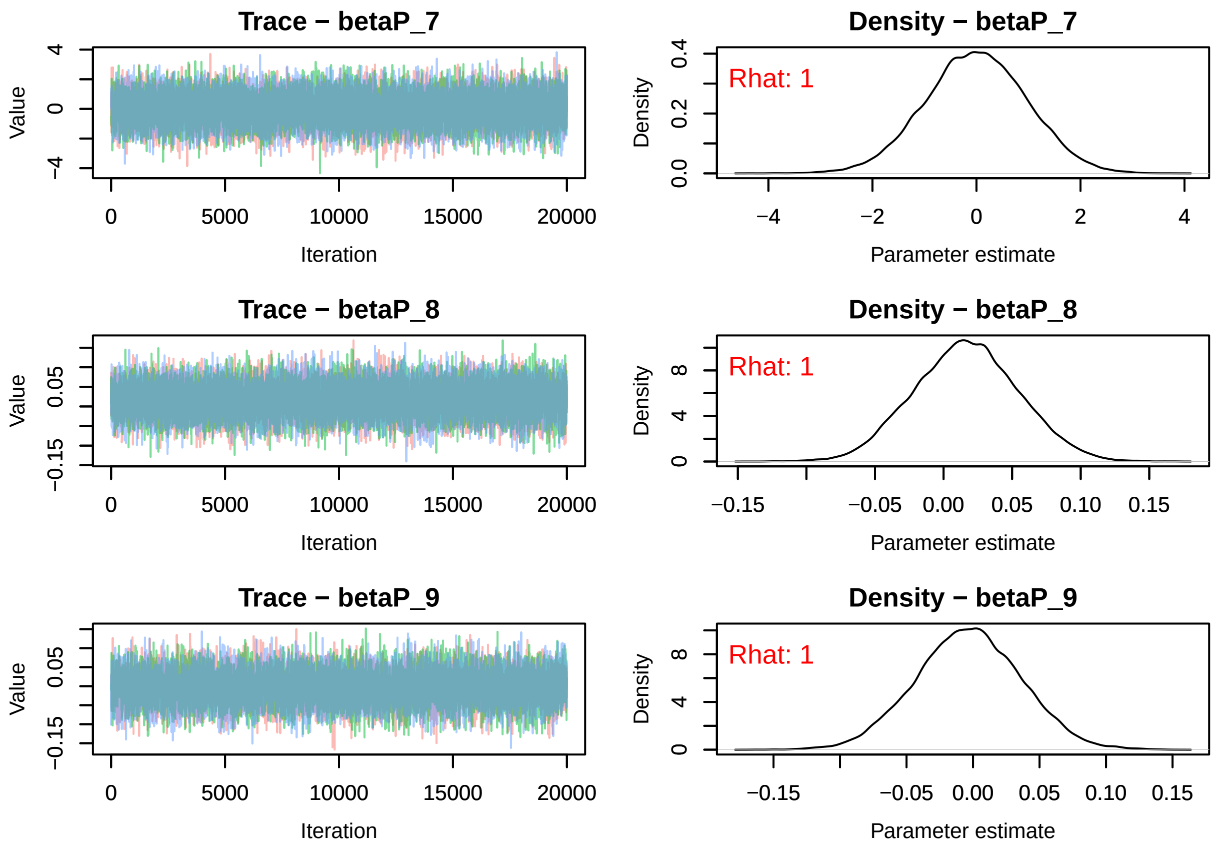
Trace − betaP_7
Density − betaP_7
Rhat: 1
Trace − betaP_8
Density − betaP_8
Rhat: 1
Trace − betaP_9
Density − betaP_9
Rhat: 1
Value
Density
Iteration
Parameter estimate

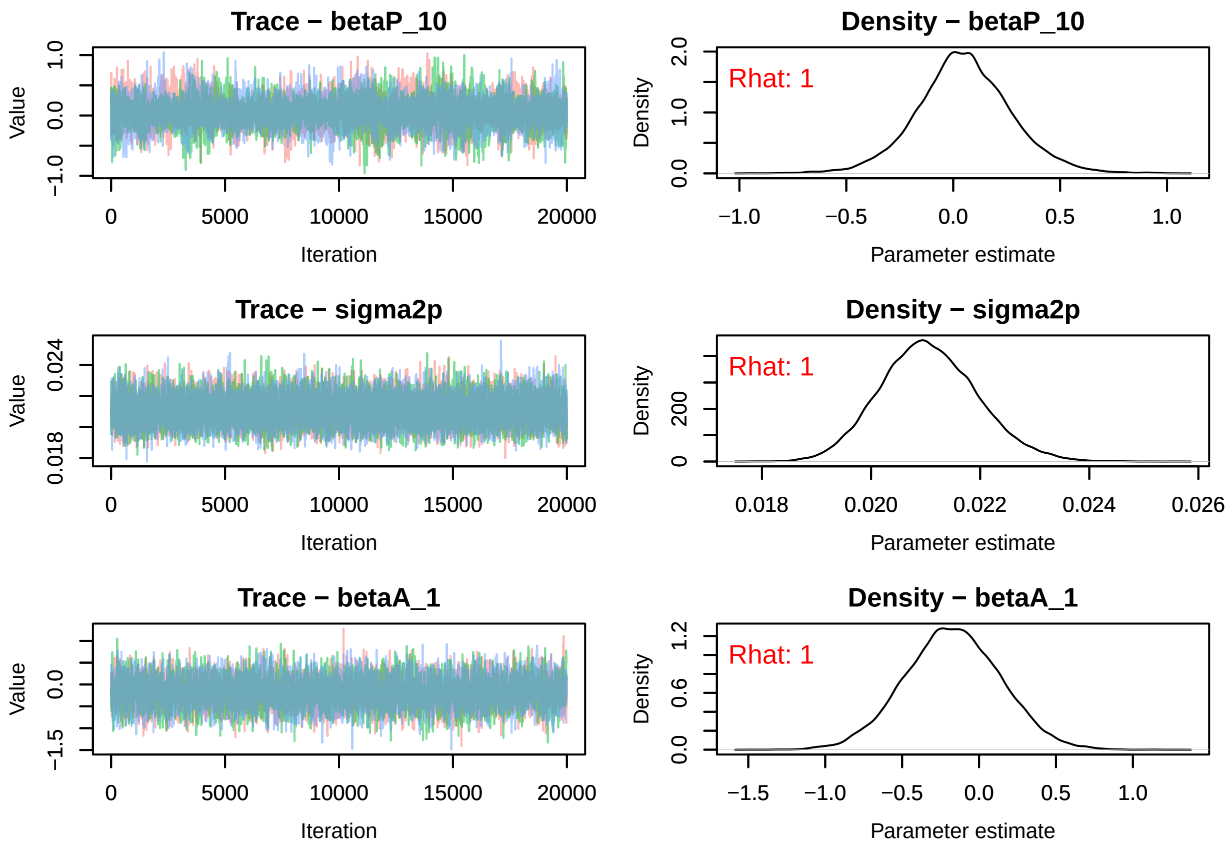
Trace − betaP_10
Density − betaP_10
Rhat: 1
Trace − sigma2p
Density − sigma2p
Rhat: 1
Trace − betaA_1
Density − betaA_1
Rhat: 1
Value
Iteration
Density
Parameter estimate

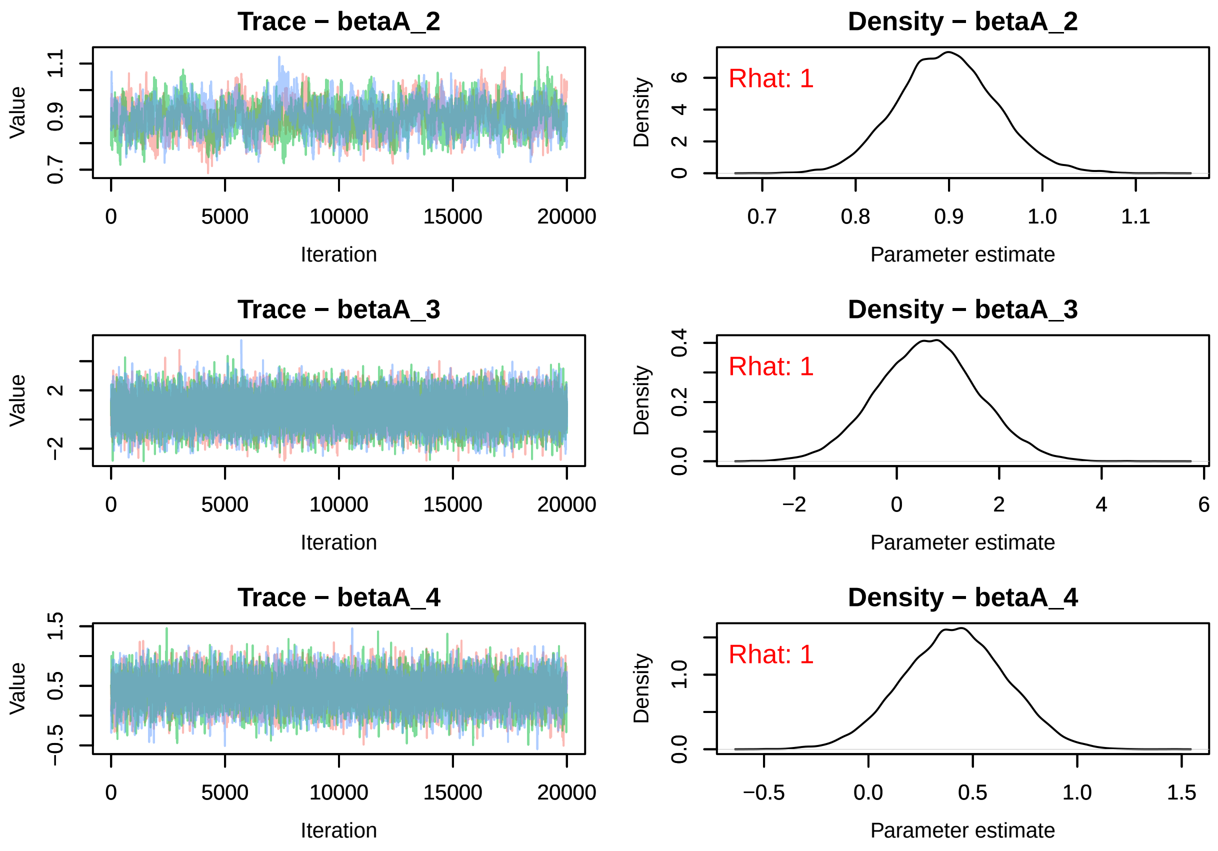
Trace − betaA_2
Density − betaA_2
Rhat: 1
Trace − betaA_3
Density − betaA_3
Rhat: 1
Trace − betaA_4
Density − betaA_4
Rhat: 1
Value
Density
Iteration
Parameter estimate

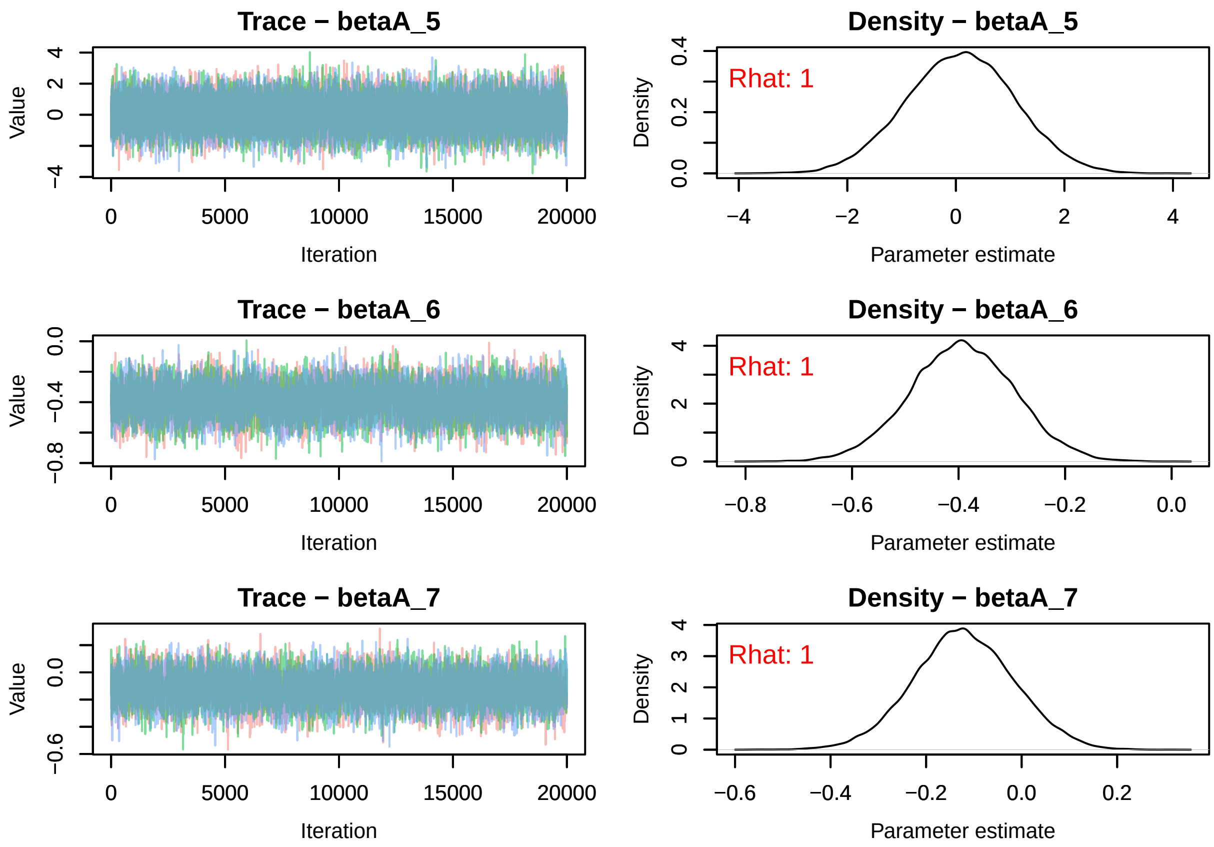
Trace − betaA_5
Value
Iteration
Density − betaA_5
Rhat: 1
Density
Parameter estimate
Trace − betaA_6
Value
Iteration
Density − betaA_6
Rhat: 1
Density
Parameter estimate
Trace − betaA_7
Value
Iteration
Density − betaA_7
Rhat: 1
Density
Parameter estimate

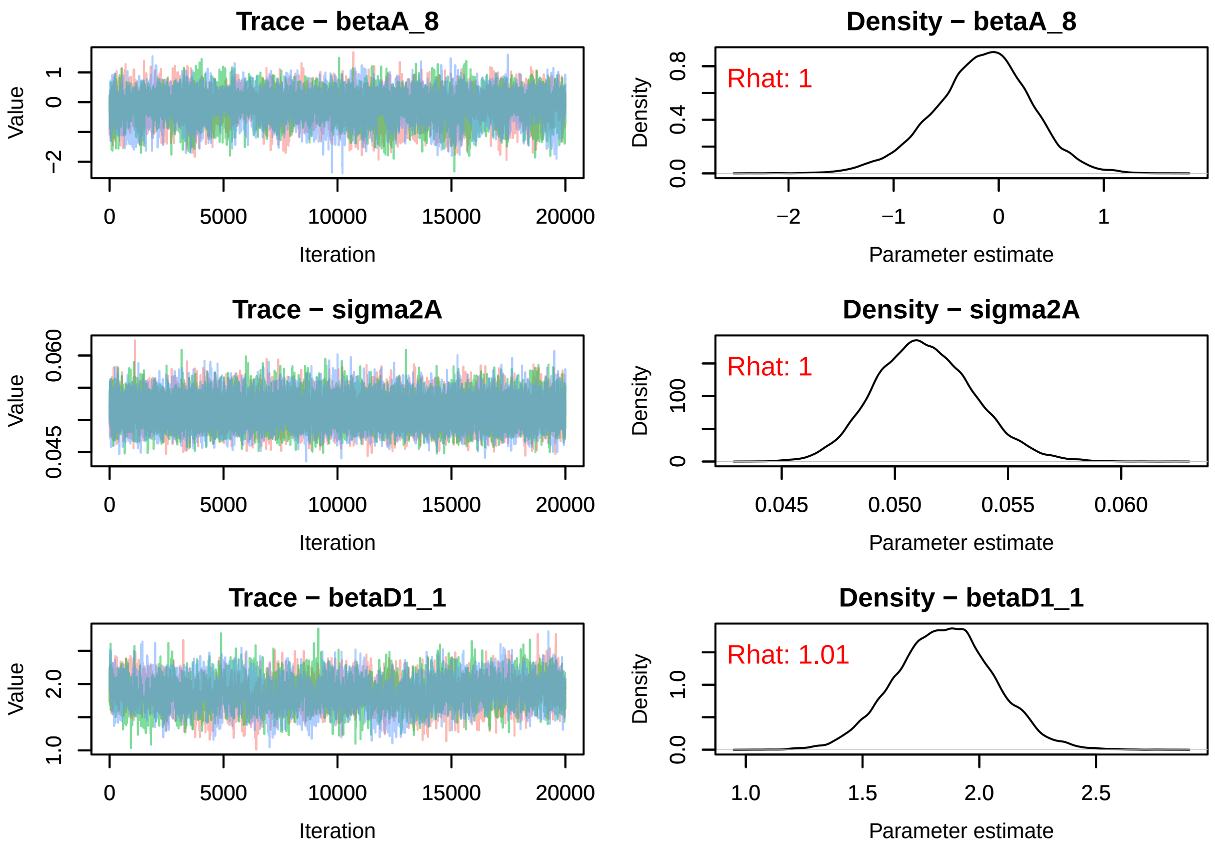

Trace − betaA_8
Density − betaA_8
Rhat: 1
Trace − sigma2A
Density − sigma2A
Rhat: 1
Trace − betaD1_1
Density − betaD1_1
Rhat: 1.01
Value
Iteration
Density
Parameter estimate

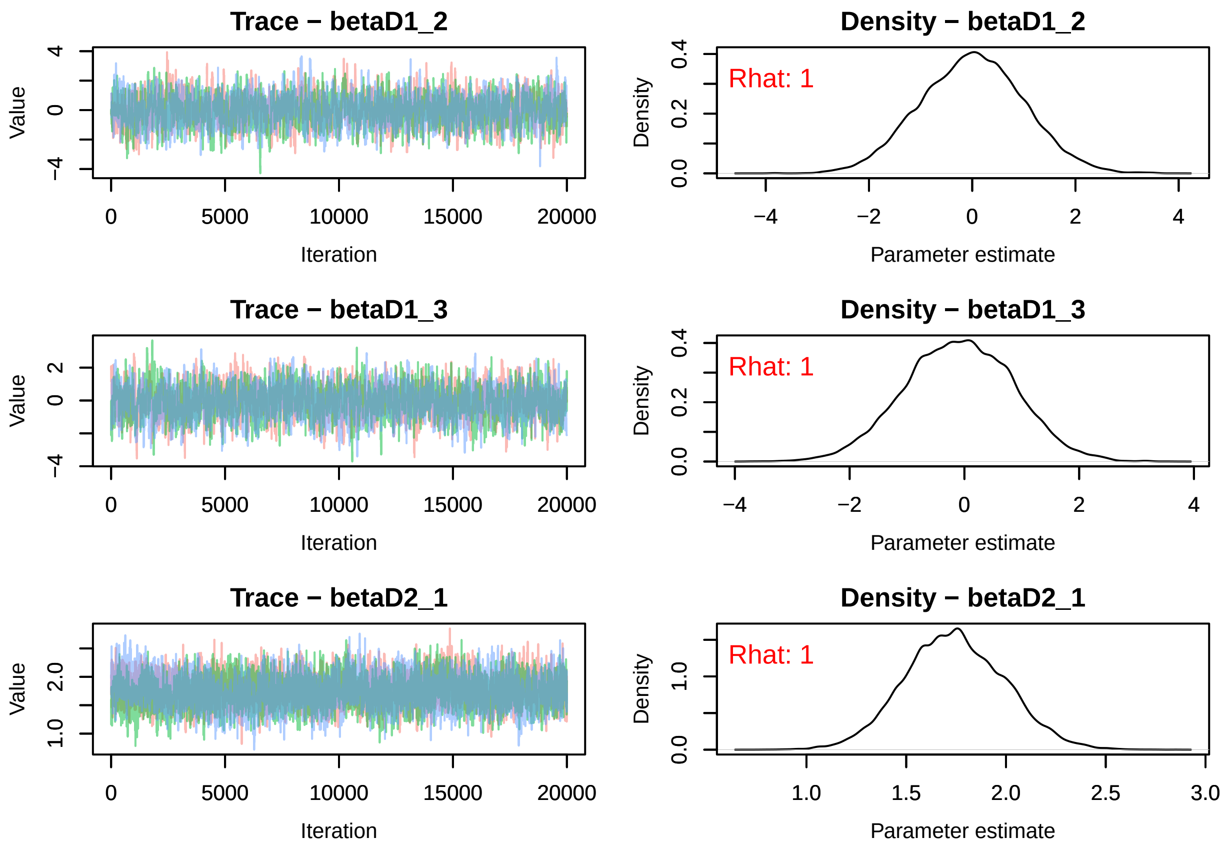

Trace − betaD1_2
Value
Iteration
Density − betaD1_2
Rhat: 1
Density
Parameter estimate
Trace − betaD1_3
Value
Iteration
Density − betaD1_3
Rhat: 1
Density
Parameter estimate
Trace − betaD2_1
Value
Iteration
Density − betaD2_1
Rhat: 1
Density
Parameter estimate

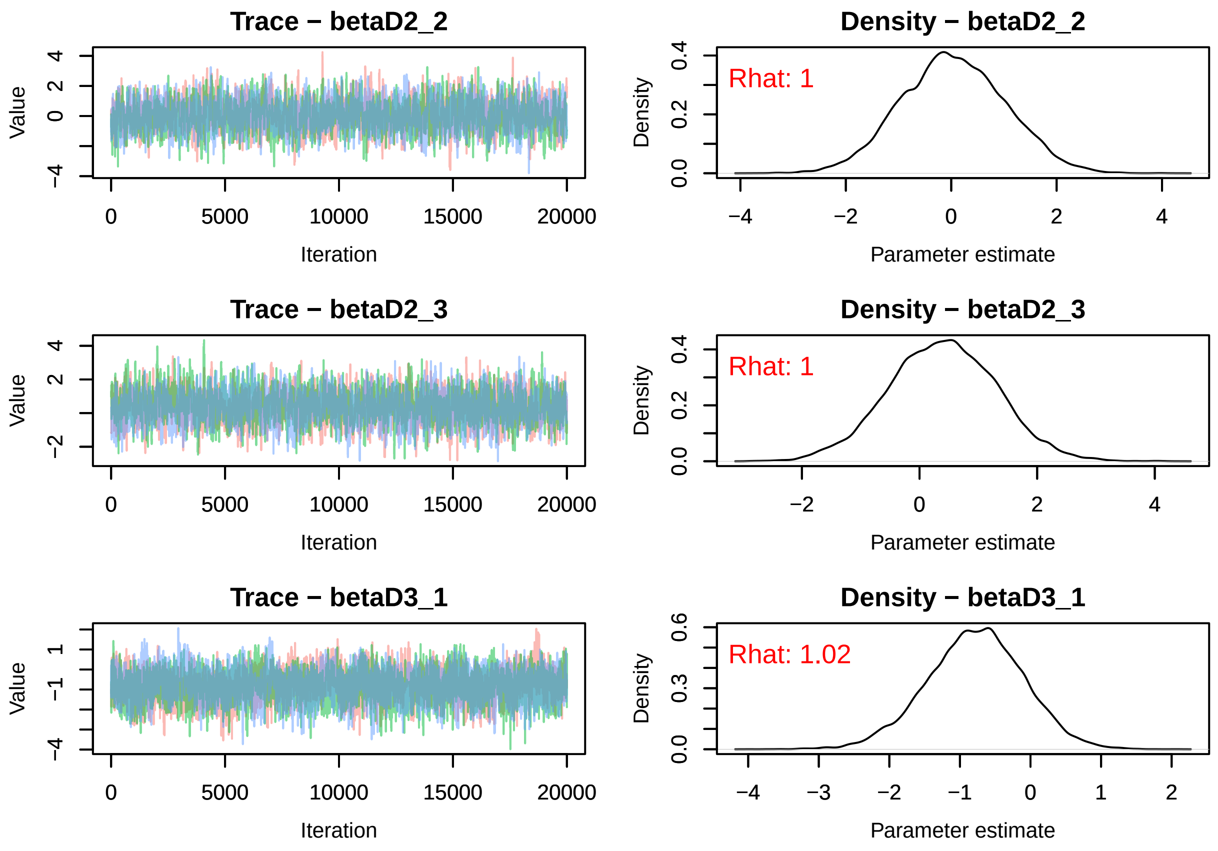
Trace − betaD2_2
Density − betaD2_2
Rhat: 1
Trace − betaD2_3
Density − betaD2_3
Rhat: 1
Trace − betaD3_1
Density − betaD3_1
Rhat: 1.02
Value
Iteration
Density
Parameter estimate

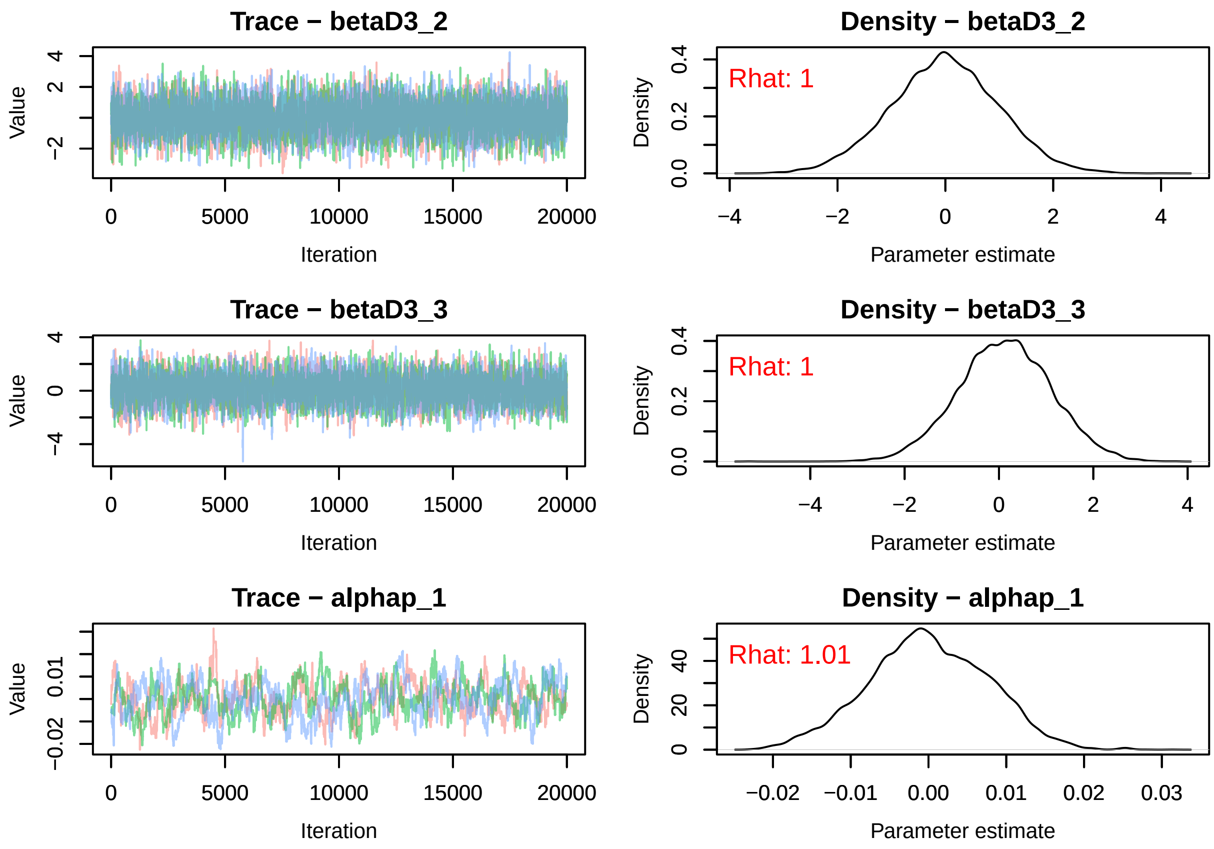
Trace − betaD3_2
Density − betaD3_2
Rhat: 1
Trace − betaD3_3
Density − betaD3_3
Rhat: 1
Trace − alphap_1
Density − alphap_1
Rhat: 1.01
Value
Iteration
Density
Parameter estimate

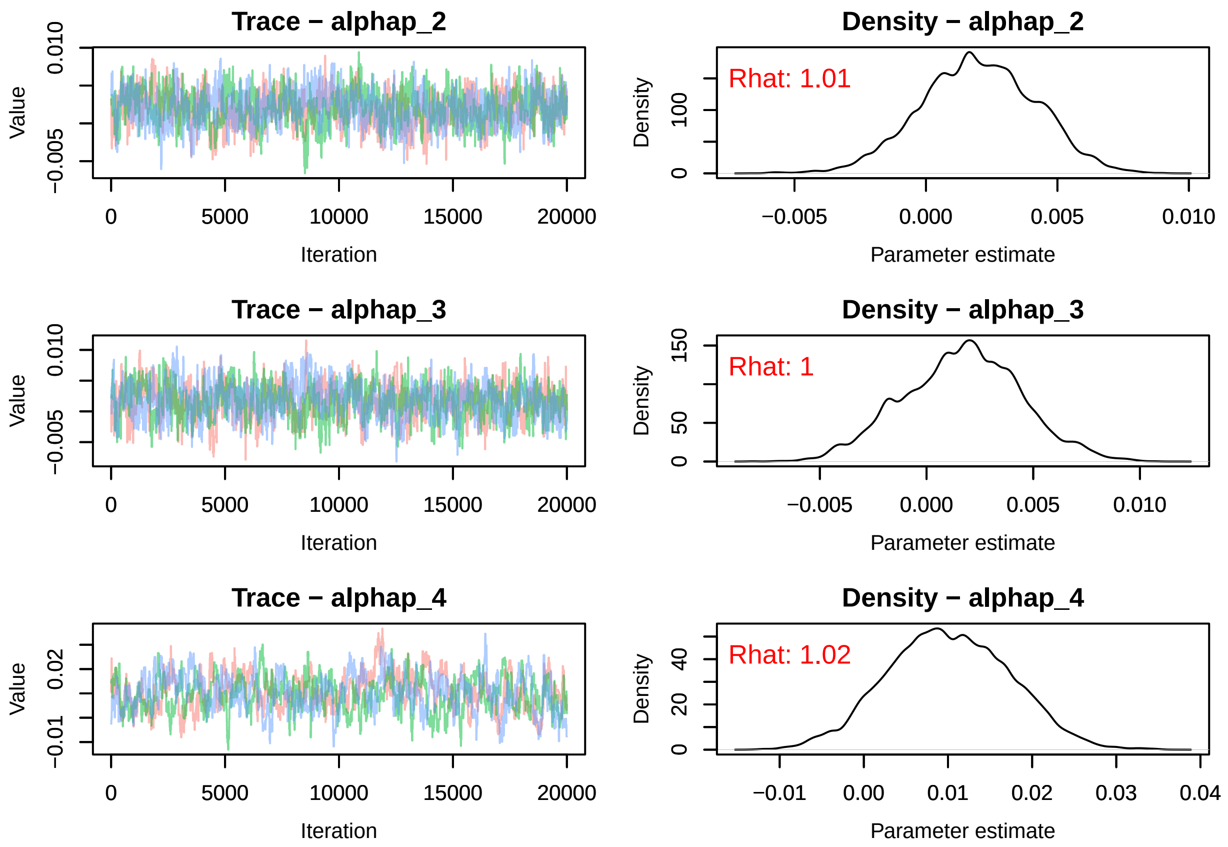
Trace − alphap_2
Density − alphap_2
Rhat: 1.01
Trace − alphap_3
Density − alphap_3
Rhat: 1
Trace − alphap_4
Density − alphap_4
Rhat: 1.02
Value
Iteration
Density
Parameter estimate

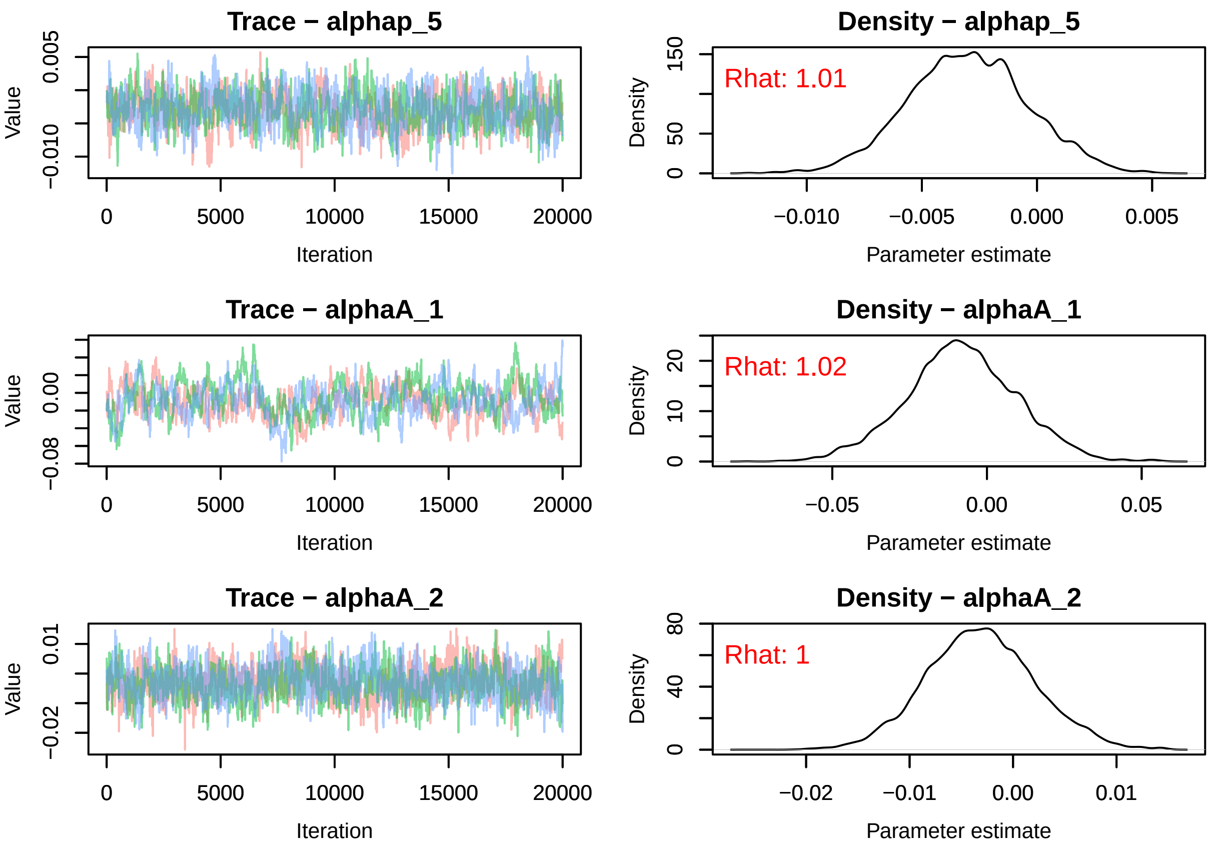
Trace − alphap_5
Value
Iteration
0
5000
10000
15000
20000
0.005
−0.010
Density − alphap_5
Density
Parameter estimate
Rhat: 1.01
150
50
0
−0.010
−0.005
0.000
0.005
Trace − alphaA_1
Value
Iteration
0.00
−0.08
Density − alphaA_1
Rhat: 1.02
20
10
−0.05
0.00
0.05
Trace − alphaA_2
Value
Iteration
0.01
−0.02
Density − alphaA_2
Rhat: 1
80
40
−0.02
−0.01
0.00
0.01

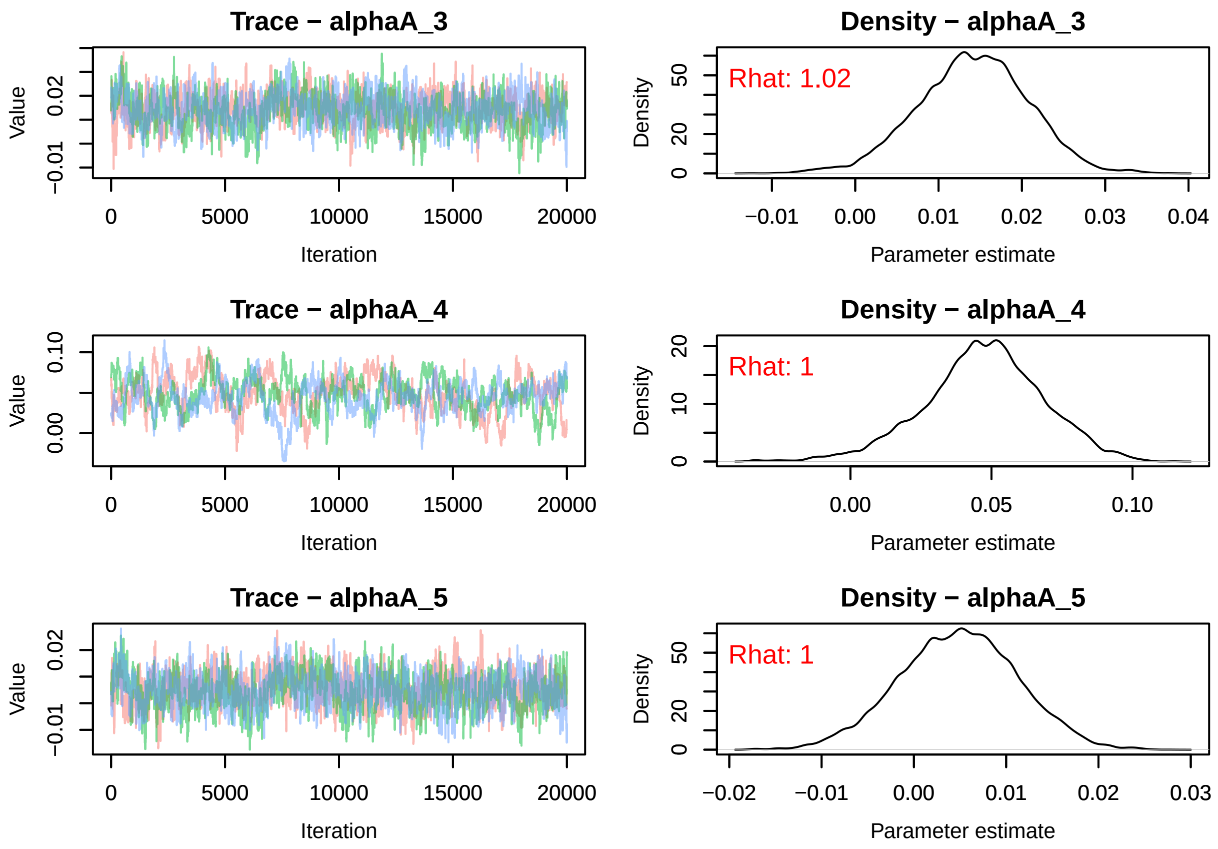
Trace − alphaA_3
Value
Iteration
Density − alphaA_3
Rhat: 1.02
Density
Parameter estimate
Trace − alphaA_4
Value
Iteration
Density − alphaA_4
Rhat: 1
Density
Parameter estimate
Trace − alphaA_5
Value
Iteration
Density − alphaA_5
Rhat: 1
Density
Parameter estimate

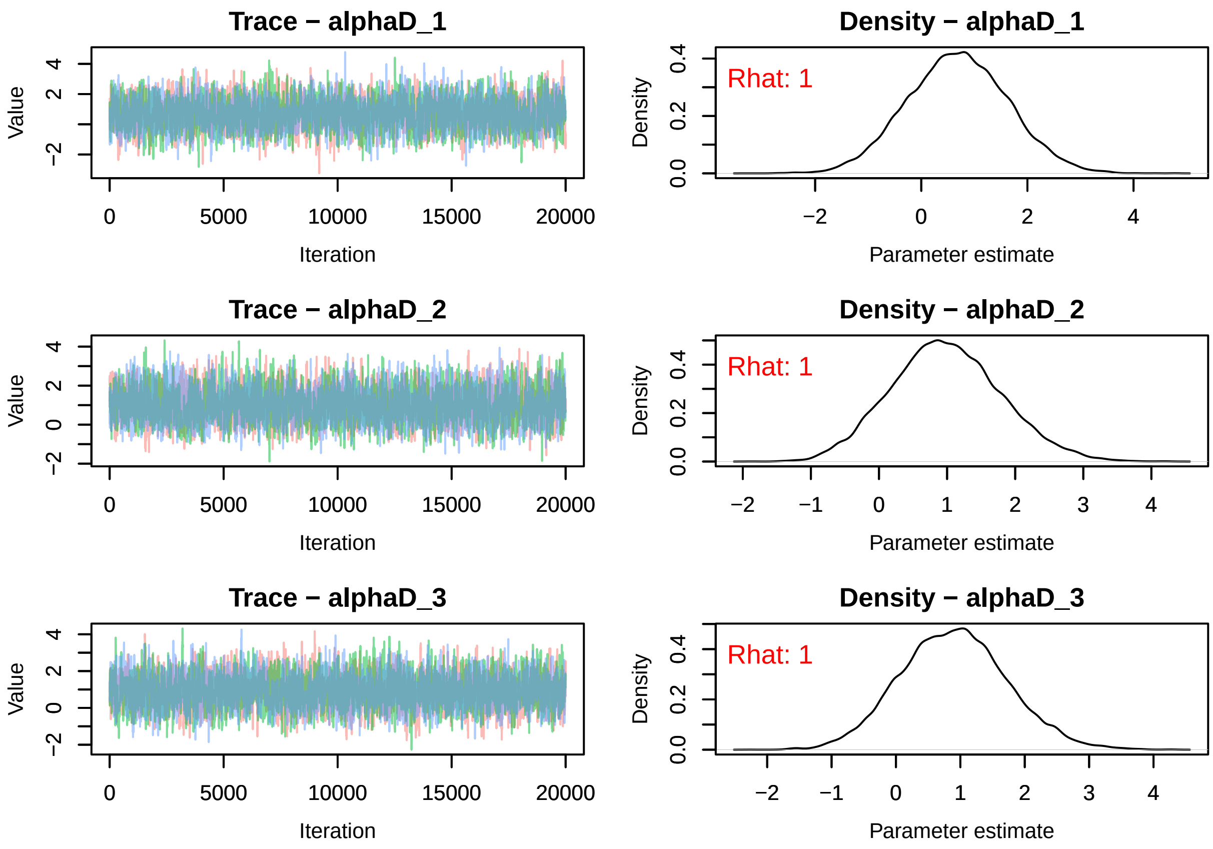
Trace − alphaD_1
Value
Iteration
Density − alphaD_1
Rhat: 1
Density
Parameter estimate
Trace − alphaD_2
Value
Iteration
Density − alphaD_2
Rhat: 1
Density
Parameter estimate
Trace − alphaD_3
Value
Iteration
Density − alphaD_3
Rhat: 1
Density
Parameter estimate

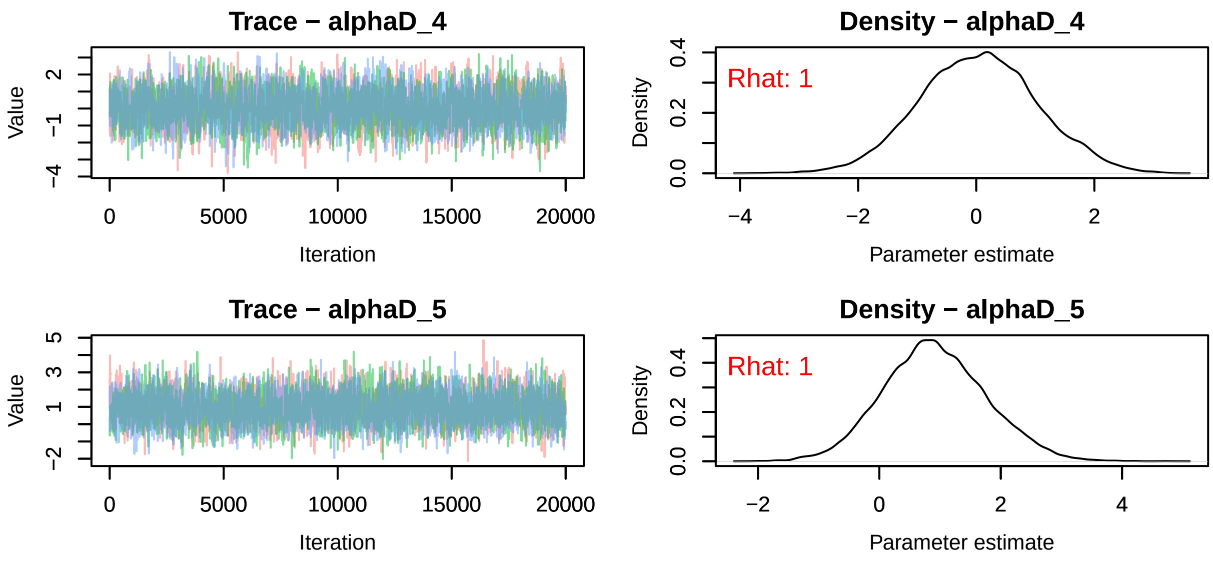
Trace − alphaD_4
Density − alphaD_4
Rhat: 1
Value
Iteration
Density
Parameter estimate
Trace − alphaD_5
Density − alphaD_5
Rhat: 1
Value
Iteration
Density
Parameter estimate
